# A recurrence model for non-puerperal mastitis patients based on machine learning

**DOI:** 10.1371/journal.pone.0315406

**Published:** 2025-01-16

**Authors:** Gaosha Li, Qian Yu, Feng Dong, Zhaoxia Wu, Xijing Fan, Lingling Zhang, Ying Yu

**Affiliations:** 1 Department of Clinical Laboratory, Affiliated Jinhua Hospital, Zhejiang University School of Medicine, Jinhua, China; 2 Department of Laboratory Medicine, The First Affiliated Hospital of Zhejiang Chinese Medical University (Zhejiang Provincial Hospital of Chinese Medicine), Hangzhou, China; 3 Department of Clinical Laboratory, Jinhua Maternal and Child Health Hospital, Jinhua, China; University of Lisbon, Institute of Social and Political Sciences, PORTUGAL

## Abstract

**Objective:**

Non-puerperal mastitis (NPM) is an inflammatory breast disease affecting women during non-lactation periods, and it is prone to relapse after being cured. Accurate prediction of its recurrence is crucial for personalized adjuvant therapy, and pathological examination is the primary basis for the classification, diagnosis, and confirmation of non-puerperal mastitis. Currently, there is a lack of recurrence models for non-puerperal mastitis. The aim of this research is to create and validate a recurrence model using machine learning for patients with non-puerperal mastitis.

**Methods:**

We retrospectively collected laboratory data from 120 NPM patients, dividing them into a non-recurrence group (n = 59) and a recurrence group (n = 61). Through random allocation, these individuals were split into a training cohort and a testing cohort in a 90%:10% ratio for the purpose of building the model. Additionally, data from 25 NPM patients from another center were collected to serve as an external validation cohort for the model. Univariate analysis was used to examine differential indicators, and variable selection was conducted through LASSO regression. A combination of four machine learning algorithms (XGBoost、Logistic Regression、Random Forest、AdaBoost) was employed to predict NPM recurrence, and the model with the highest Area Under the Curve (AUC) in the test set was selected as the best model. The finally selected model was interpreted and evaluated using Receiver Operating Characteristic (ROC) curves, calibration curves, Decision curve analysis (DCA), and Shapley Additive Explanations (SHAP) plots.

**Results:**

The logistic regression model emerged as the optimal model for predicting recurrence of NPM with machine learning, primarily utilizing three variables: FIB, bacterial infection, and CD4^+^ T cell count. The model showed an AUC of 0.846 in the training cohort and 0.833 in the testing cohort. The calibration curve indicated excellent calibration of the model. DCA revealed that the model possessed favorable clinical utility. Furthermore, the model effectively achieved in the external validation group, with an AUC of 0.825.

**Conclusion:**

The machine learning model developed in this study, serving as an effective tool for predicting NPM recurrence, aids doctors in making more individualized treatment decisions, thereby enhancing therapeutic efficacy and reducing the risk of recurrence.

## Introduction

Non-puerperal mastitis is a rare, non-malignant condition that affects the breast, making up about 4–5% of all benign breast lesions [1]. It mainly consists of periductal mastitis and granulomatous mastitis [2]. NPM mainly affects women aged 30–40 with a history of childbearing [3–6]. NPM mostly occurs unilaterally, manifesting as breast masses, which can later develop into abscesses, sinus tracts, or ulcers. It is typically accompanied by symptoms such as redness, swelling, warmth, and discomfort in the breast, often resulting in a prolonged and recurrent course of the disease [7,8]. The clinical manifestations of NPM are similar to those of breast cancer, making it prone to confusion. Clinicians often use imaging examinations, including ultrasound, mammography, and magnetic resonance imaging, to aid in the diagnosis of NPM. Histopathology serves as the gold standard for diagnosis [9]. Apart from breast inflammation, NPM may also be accompanied by systemic symptoms such as joint swelling and pain, nodular erythematosus on the extremities, and scleritis [10–13]. In recent years, the clinical incidence of NPM has risen significantly, with the most notable increase observed in China [14]. Among all existing studies, the recurrence rate of NPM can range from 4% to 65% [15], with an average of 17% of patients experiencing recurrence [16]. The long-term treatment and recurrent episodes of the disease cause immense suffering for patients, damaging the appearance of the breasts and severely impacting the mental health and quality of life of young women. Therefore, it is highly necessary to establish models to predict whether NPM patients will experience recurrence, providing guidance for identifying recurrence risks in clinical treatment plans.

Machine learning, the core of artificial intelligence, utilizes complex algorithms to assist humans in solving problems. Its theories and methods have been widely applied in the medical field. Through machine learning, individual characteristics of data can be identified, and models can be scientifically established. These models can then be used to predict future data based on new information [17]. Currently, there is a study that employs a scoring system, incorporating criteria such as having more than two births, breastfeeding for over 18 months, and a BMI exceeding 30 kg/m^2^, to predict the recurrence of NPM [18]. Presently, there is only one study that has developed a machine learning-based model for predicting the recurrence of non-puerperal mastitis, and it only incorporates a limited amount of laboratory test data, including white blood cell count (WBC), neutrophil-to-lymphocyte ratio (NLR), albumin-to-globulin ratio (AGR), and triglycerides [19]. The use of objective hematological indicators and differential diagnostic markers for distinguishing between different diseases has been a focal point in various research studies. Exploring the potential relationships between these markers and illness could reveal more straightforward and reliable clinical indicators. Therefore, this study has established a machine learning-based model for predicting NPM recurrence by analyzing various clinical laboratory test results, enabling clinicians to identify potential recurrent patients as early as possible.

Listed below are the key findings of this research: 1. A new model based on machine learning has been established to predict the recurrence of non-puerperal mastitis. 2. This present study compares the predictive capabilities of four different machine learning algorithms for NPM recurrence among patients, with the logistic regression model demonstrating the highest predictive accuracy.

## Methods

### Ethical statement

This study was approved by the Ethics Committee of the First Affiliated Hospital of Zhejiang Chinese Medical University, with the ethics approval number 2024-KL-337-01.

### Patient involvement

We retrospectively collected data from 146 NPM patients who initially presented at the Hubin Center of the First Affiliated Hospital of Zhejiang Chinese Medical University between January 1, 2022, and July 30, 2023. And we accessed the relevant data of these patients by reviewing the electronic medical record system from 30 April 2024 to 5 May 2024. Inclusion criteria for patients: (1) Pathological examination results of breast masses, obtained through fine-needle aspiration, core needle biopsy, or surgery, support a diagnosis of non-puerperal mastitis; (2) Female patients aged 18 to 50 years. Exclusion criteria for patients: (1) Patients who are pregnant and breastfeeding; (2) Patients with breast cancer or breast tuberculosis; (3) patients with serious illnesses such as heart disease, diabetes, and uremia. Based on the inclusion and exclusion criteria, a total of 120 patients were ultimately included in this study, and their data will be used to establish the model. Additionally, this study included 25 NPM patients from the Qiantang Center of the First Affiliated Hospital of Zhejiang Chinese Medical University, and their data were used for external validation of the model.

According to the Expert Consensus on Traditional Chinese Medicine Diagnosis and Treatment of Granulomatous Lobular Mastitis (2021 Edition) [20], the clinical cure is defined as incomplete imaging remission, the disappearance of clinical symptoms, inability to palpate the original inflammatory lesion, healing of ulcers or wounds, but the presence of scattered small lesions still visible on imaging. After achieving the clinical cure standard for the original lesion in the breast and following up for a continuous period of up to six months, the recurrence will be considered if symptoms such as redness, swelling, heat, pain, abscess formation, or ulceration reappear locally at the site of the lesion. Based on the definition of recurrence, the 120 patients were divided into a non-recurrence group (n = 59) and a recurrence group (n = 61). Among the 25 NPM patients used for external validation, there were 18 in the non-recurrence group and 7 in the recurrence group.

### Data collection

Baseline information (age, BMI, reproductive history) and pre-treatment clinical laboratory data were retrieved from the electronic medical records of these patients. The laboratory data encompassed a total of 57 indicators, including bacterial culture, routine blood examination, reproductive hormone tests, immune-related tests, and coagulation tests.

### Data analysis

The collected data were analyzed and processed using SPSS version 26.0 and R version 4.3.1 software. Categorical variables were analyzed using the chi-square test and presented as frequencies and percentages. Numerical variables were analyzed using either the independent samples t-test or the Wilcoxon test, depending on the normality of distribution and homogeneity of variances, and presented as medians with interquartile ranges (IQR). A *P*-value < 0.05 was considered statistically significant. Subsequently, LASSO regression was applied to further screen the variables with significant differences, and logistic regression was used to select the variable set.

### Machine learning

**Fig 1:**
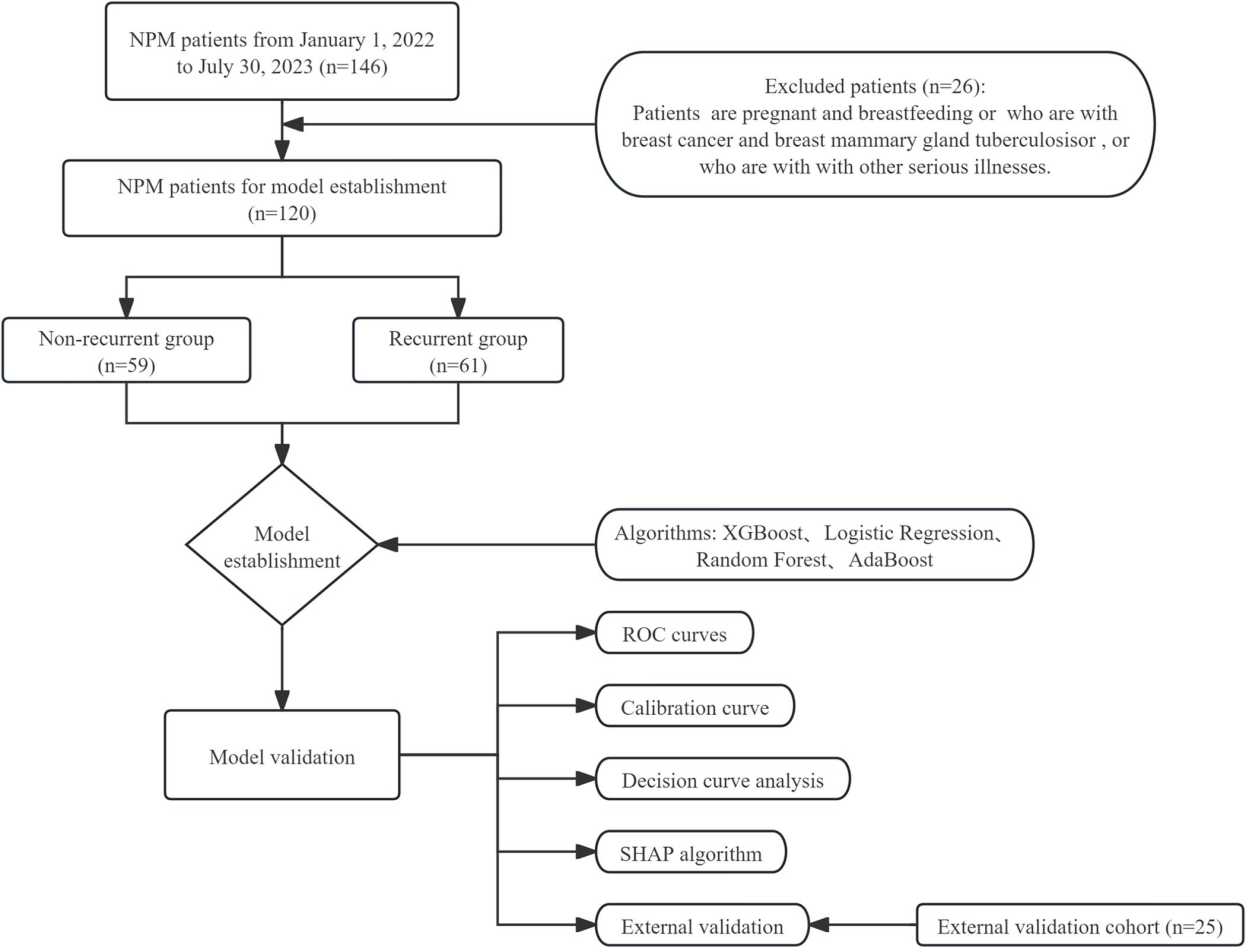
The flow chart for the study.

We employed the Deepwise & Beckman Coulter DxAI platform for model comparison, establishment, and evaluation. 120 patients were randomly allocated into training (90%) and testing (10%) cohorts, with a fixed random seed (random seed = 20). Four different models (XGBoost, Logistic Regression, Random Forest, AdaBoost) were evaluated using 5-fold cross-validation. The model with the highest AUC in the test cohort was selected as the best model among the four models. In order to evaluate the model’s performance, we utilized measurements such as accuracy, sensitivity, specificity, ROC, AUC, DCA, and calibration curves. Additionally, we utilized SHAP plots to interpret the best model, visualizing the model to facilitate a better understanding. The entire methodological process of our study is illustrated in Fig 1.

## Results

### Baseline data

The experimental group’s demographic characteristics are outlined in Table 1. The comparison between the two groups revealed no statistically meaningful differences in terms of age and BMI (*P*>0.05). However, the BMI of both groups exceeded the normal reference range. The proportion of patients with a reproductive history in the recurrence group was significantly higher than that in the non-recurrence group (*P*<0.05).

**Table 1:** Patient characteristics of non-puerperal mastitis.

Variable	Group		*P*-value^*2*^
	No recurrence, N = 59	Recurrence, N = 61	
**Age** **[year (median IQR)]**	32.000 (28.000–34.500)	32.000 (30.000–34.000)	0.502
**BMI** **[kg/m^2 (median IQR)]**	24.133 (23.388–25.813)	24.331 (22.833–25.609)	0.659
**Reproductive history**			0.003
** No [(n, %)]**	14 (24)	3 (4.9)	
**Yes [(n, %)]**	45 (76)	58 (95)	

### Comparison of clinical laboratory data between non-recurrence and recurrence groups of NPM patients

In this study, we compared 57 laboratory indicators from 120 NPM patients. As Table 2 illustrates, prominent differences (*P*<0.05) among the non-recurrence and recurrence groups were evident in 13 indicators: bacterial infection, WBC, NE, MO, PDW, NLR, PLR, CRP, IL-6, CD4^+^ T cell count, B cell count, FIB, and DD. Specifically, the recurrence group exhibited significantly higher rates of bacterial infection, WBC, NE, MO, PDW, NLR, PLR, CRP, IL-6, FIB, and DD compared to the non-recurrence group. Conversely, the non-recurrence group had noteworthy higher CD4^+^ T cell and B cell counts than the recurrence group.

**Table 2:** Comparison of clinical laboratory data between non-recurrence group and recurrence group.

Variable	Group		*P*-value^*2*^
	No recurrence, N = 59	Recurrence, N = 61	
**Bacterial infection**			<0.001
**No [(n, %)]**	55 (93)	39 (64)	
**Yes [(n, %)]**	4 (6.8)	22 (36)	
**WBC (×10** ^**9**^ **/L)**	8.804 (7.600–9.950)	9.900 (8.438–11.900)	0.009
**NE (×10** ^**9**^ **/L)**	6.100 (4.850–6.600)	6.400 (5.700–8.925)	0.001
**LY (×10** ^**9**^ **/L)**	2.000 (1.600–2.500)	1.700 (1.300–2.100)	0.052
**MO (×10** ^**9**^ **/L)**	0.500 (0.300–0.600)	0.600 (0.400–0.700)	0.023
**EO (×10** ^**9**^ **/L)**	0.100 (0.055–0.150)	0.090 (0.060–0.120)	0.383
**BA (×10** ^**9**^ **/L)**	0.030 (0.010–0.050)	0.020 (0.010–0.040)	0.955
**RBC (×10** ^**12**^ **/L)**	4.440 (4.175–4.725)	4.340 (4.050–4.550)	0.084
**HGB (g/L)**	129.000 (121.500–135.000)	126.000 (120.000–135.000)	0.372
**HCT (%)**	38.900 (36.600–40.660)	37.600 (35.600–40.100)	0.167
**MCH (pg)**	29.100 (27.150–29.950)	29.100 (28.200–30.200)	0.308
**MCHC (g/L)**	333.000 (327.000–337.500)	333.000 (328.000–341.000)	0.327
**MCV (fl)**	86.700 (84.200–89.300)	87.200 (84.400–89.900)	0.497
**RDW (%)**	12.700 (12.200–13.500)	12.900 (12.400–13.400)	0.612
**PLT (×10** ^**9**^ **/L)**	288.000 (250.000–337.000)	299.000 (249.000–370.000)	0.434
**PCT (%)**	0.253 (0.227–0.283)	0.254 (0.226–0.302)	0.257
**MPV (fl)**	8.700 (7.750–9.800)	8.700 (7.900–10.100)	0.523
**PDW (%)**	16.100 (15.800–16.400)	16.200 (16.000–16.500)	0.035
**NLR**	3.000 (2.248–3.997)	4.143 (3.188–5.667)	<0.001
**PLR**	138.889 (111.000–198.235)	181.765 (143.000–212.105)	0.016
**CRP (mg/L)**	5.300 (1.415–12.465)	15.820 (7.380–40.750)	<0.001
**FSH (IU/L)**	4.880 (3.790–5.770)	4.922 (3.820–6.340)	0.885
**LH (IU/L)**	4.144 (2.740–6.734)	4.150 (2.710–7.080)	0.840
**PRL (mIU/L)**	450.660 (289.128–644.469)	429.907 (264.850–615.776)	0.443
**E2 (pmol/L)**	196.137 (153.620–364.755)	214.004 (177.130–356.210)	0.358
**T (nmol/L)**	1.080 (0.836–1.405)	1.050 (0.810–1.182)	0.150
**P (nmol/L)**	1.498 (0.545–6.240)	1.860 (0.610–12.771)	0.218
**IgA (g/L)**	2.660 (2.145–3.135)	2.640 (2.020–3.430)	0.757
**IgG (g/L)**	12.800 (11.640–14.900)	12.900 (11.672–14.200)	0.801
**IgM (g/L)**	1.621 (1.290–2.065)	1.580 (1.230–2.010)	0.571
**C3 (g/L)**	1.142 (1.015–1.240)	1.200 (1.030–1.300)	0.201
**C4 (g/L)**	0.260 (0.215–0.290)	0.270 (0.230–0.340)	0.216
**IL-2 (pg/ml)**	1.300 (1.067–1.583)	1.340 (1.190–1.610)	0.461
**IL-4 (pg/ml)**	1.270 (0.804–1.572)	1.130 (0.900–1.530)	0.823
**IL-6 (pg/ml)**	4.392 (3.225–6.150)	6.520 (4.540–11.729)	<0.001
**IL-10 (pg/ml)**	2.278 (1.797–2.790)	2.460 (1.907–3.010)	0.402
**TNF-α (pg/ml)**	1.270 (0.830–2.655)	1.223 (1.008–2.620)	0.761
**IFN-γ (pg/ml)**	1.300 (1.155–2.250)	1.450 (1.190–2.430)	0.293
**CD4** ^**+**^ **CD25** ^**+**^ **T cells (%)**	2.400 (1.825–3.300)	2.500 (2.000–3.400)	0.461
**Treg cells (%)**	6.120 (5.600–7.725)	6.000 (5.100–7.700)	0.459
**CD45** ^**+**^ **T cells (%)**	73.560 (67.553–76.000)	74.003 (68.030–77.850)	0.362
**CD4** ^**+**^ **T cells (%)**	39.000 (34.495–44.325)	40.557 (36.520–45.220)	0.378
**CD8** ^**+**^ **T cells (%)**	26.480 (22.290–29.260)	26.300 (22.070–30.220)	0.910
**CD4** ^**+**^ **T cells /CD8** ^**+**^ **T cells**	1.452 (1.220–1.935)	1.500 (1.180–1.890)	0.842
**B cells (%)**	13.440 (11.245–15.650)	12.921 (10.930–14.800)	0.454
**NK cells (%)**	10.330 (8.605–14.780)	10.410 (7.670–16.470)	0.937
**CD45** ^**+**^ **T cells (×10** ^**6**^ **/L)**	1,222.000 (997.500–1,530.000)	1,118.710 (878.000–1,294.000)	0.088
**CD4** ^**+**^ **T cells (×10** ^**6**^ **/L)**	656.000 (509.000–877.000)	595.640 (430.000–753.000)	0.045
**CD8** ^**+**^ **T cells (×10** ^**6**^ **/L)**	481.030 (337.000–608.000)	403.000 (294.000–502.000)	0.090
**B cells (×10** ^**6**^ **/L)**	243.910 (158.500–331.500)	200.000 (147.000–267.000)	0.044
**NK cells (×10** ^**6**^ **/L)**	165.000 (128.000–253.500)	149.000 (105.070–237.000)	0.168
**PT (s)**	11.800 (11.100–12.100)	11.800 (11.500–12.100)	0.206
**INR**	0.980 (0.930–1.020)	0.990 (0.960–1.030)	0.147
**FIB (g/L)**	3.830 (3.240–4.386)	5.300 (4.290–5.950)	<0.001
**TT (s)**	17.500 (16.950–18.100)	17.400 (16.800–17.900)	0.260
**APTT (s)**	28.764 (27.550–29.550)	28.700 (27.500–30.700)	0.392
**DD (mg/L)**	0.310 (0.200–0.540)	0.550 (0.310–0.820)	0.004

WBC, leukocyte count; NE, neutrophil; LY, lymphocyte; MO, monocyte; EO, eosinophil; BA, basophil; RBC, red cell count; HGB, hemoglobin; HCT, hematocrit; MCV, average red blood cell volume; MCH, average red blood cell hemoglobin content; MCHC, average red blood cell hemoglobin concentration; RDW, red blood cell volume distribution width; PLT, platelet; MPV, average platelet volume; PDW, platelet volume distribution width. NLR, neutrophil to lymphocyte ratio; PLR, platelet to lymphocyte ratio; CRP, C reactive protein; FSH, follicle-stimulating hormone; LH, Luteinizing hormone; PRL, prolactin; E2, estradiol; P, progesterone; T, testosterone; IG, immune globulin; C3, complement3; C4, complement4; IL, interleukin; TNF-α, tumor necrosis factorα; IFN-γ, interferon γ; CD, Cluster of differentiation antigen; Treg cell, regulatory T cell; PT, prothrombin time; INR, International Normalized Ratio; FIB, fibrinogen; TT, thrombin time; APTT, activated partial thromboplastin time; DD, D-dimer.

### LASSO regression and ROC

**Fig 2:**
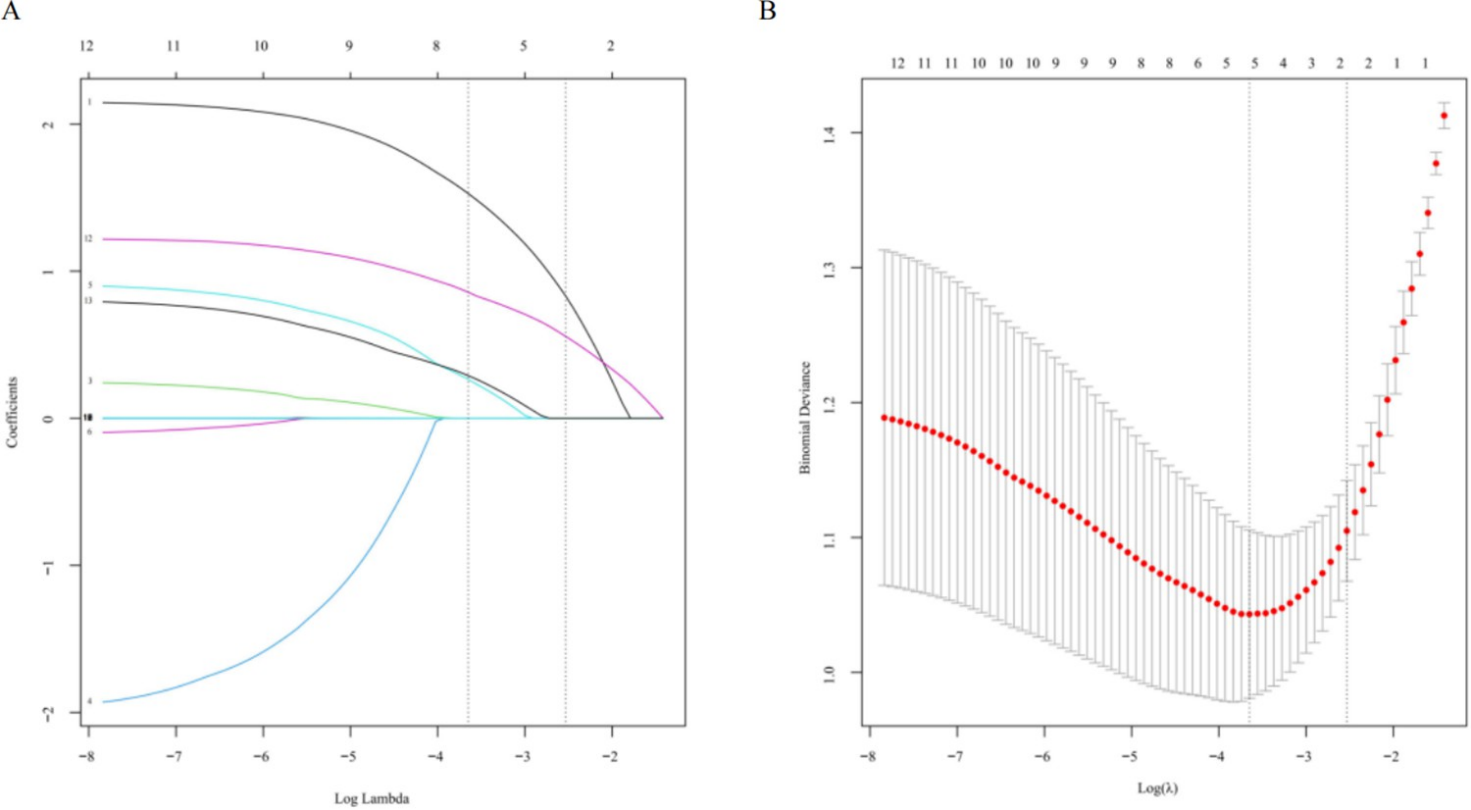
Predictors selection using LASSO regression analysis and 5-fold cross-validation. (A) Bias selection of the tuning parameter (lambda) in LASSO regression based on the minimum standard (left dashed line) and 1-SE standard (right dashed line). (B) A joint plot was created based on the loglikelihood. In this study, the selection of predictive factors was based on the 1-SE standard (left dashed line), resulting in the selection of six non-zero factors. LASSO, least absolute shrinkage and selection operator; SE, the standard error.

**Fig 3:**
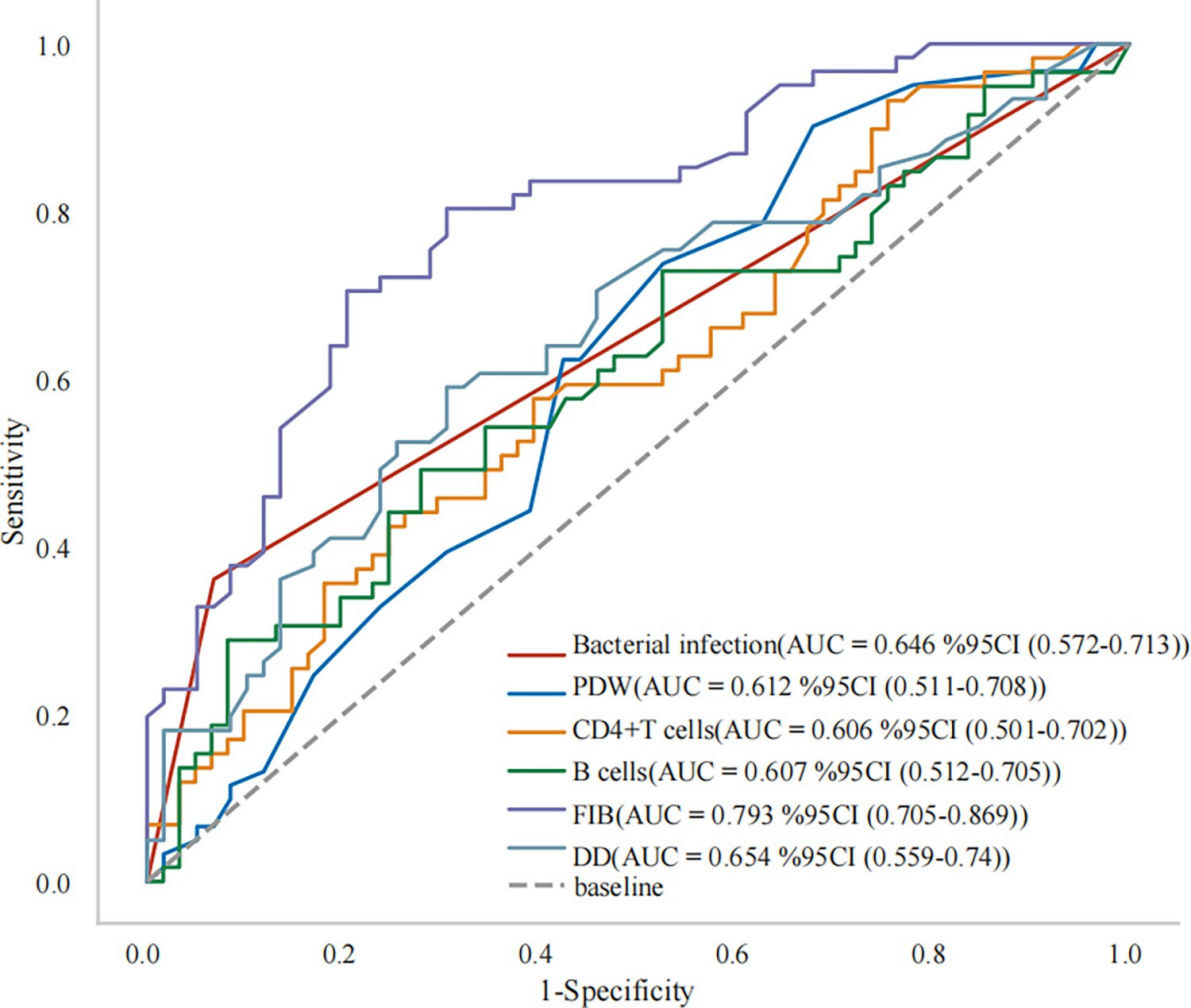
ROC curve of six impact indicators.

After eliminating irrelevant and redundant features, this study conducted a Lasso regression analysis on 13 differential indicators. Lasso regression employs a penalty function to compress the variable coefficients. This compression technique aims to prevent overfitting and address the issue of severe collinearity, thereby enhancing the model’s predictive accuracy and interpretability. The results showed that bacterial infection, PDW, CD4^+^ T cell count, B cell count, FIB, and DD were considered relevant factors influencing NPM recurrence as shown in Fig 2. Subsequently, we conducted ROC analysis on the six obtained indicators. As can be seen in Fig 3, FIB exhibited the highest diagnostic efficiency (AUC = 0.793), succeeded by DD (AUC = 0.654) and bacterial infection (AUC = 0.646).

### Variable set selection

**Fig 4:**
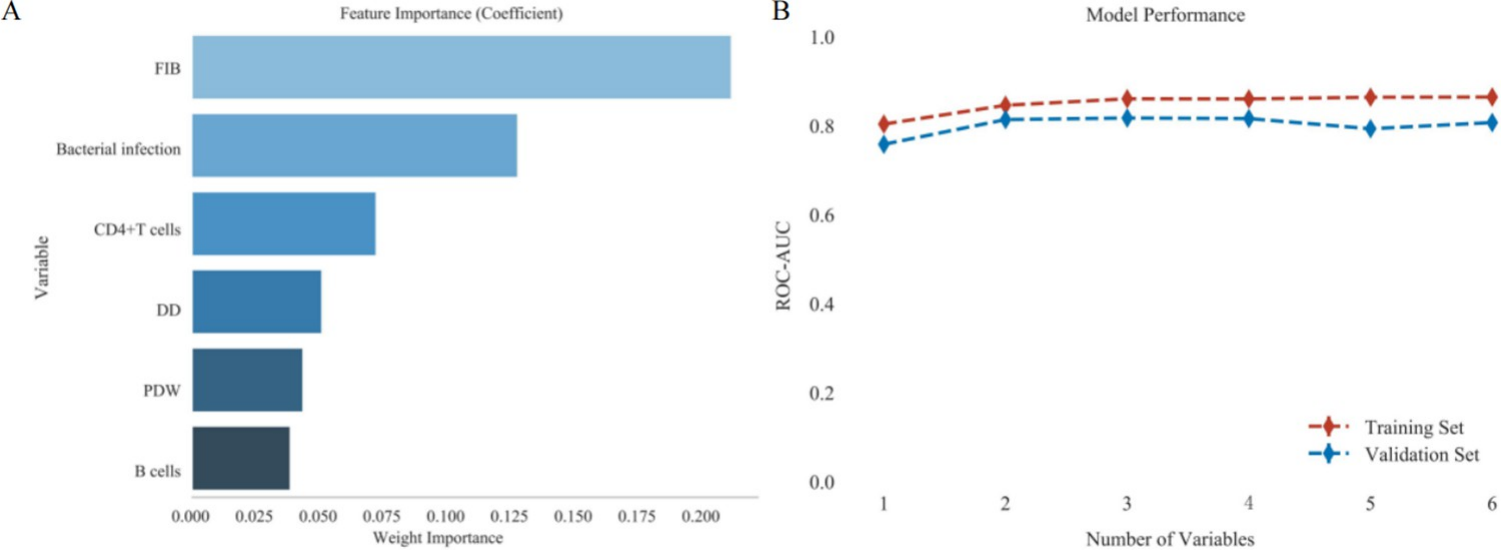
Set of variables VS model performance.

The six indicators identified through the Lasso regression analysis were ranked according to their importance, and they were added to the variable set based on this order of importance. Fig 4A illustrates the ranking of the six indicators based on their significance. The logistic regression was then employed to evaluate the performance of the variable sets. As shown in Fig 4B, the model exhibited the best performance when the variable set contained three indicators. Therefore, FIB, bacterial infection, and CD4^+^T cell count were selected as the final indicators to be included in the model.

### Comparison of the four models and selection of the optimal model

Table 3 presents the AUC values for four machine learning algorithms obtained through 5-fold cross-validation. In the testing cohort, the outcomes of four distinct machine learning algorithms revealed that XGBoost had an AUC of 0.664, Logistic Regression had an AUC of 0.833, Random Forest had an AUC of 0.827, and AdaBoost had an AUC of 0.818. The logistic regression model exhibited the highest predictive capability. Additionally, the logistic regression model achieved AUC, accuracy, sensitivity, specificity, positive predictive value, negative predictive value, and F1 score values exceeding 70%.

**Table 3:** Comparison of the four models.

Classifier	Cohorts	AUC	Accuracy	Sensitivity	Specificity	Positive predictive value	Negative predictive value	F1
**XGBoost**	Training	0.860	0.787	0.815	0.788	0.811	0.775	0.812
	Testing	0.664	0.650	0.527	0.917	0.737	0.594	0.610
**Logistic** **Regression**	Training	0.846	0.789	0.809	0.792	0.797	0.783	0.803
	Testing	0.833	0.750	0.833	0.833	0.800	0.714	0.816
**Random Forest**	Training	1.000	0.991	1.000	1.000	1.000	0.982	1.000
	Testing	0.827	0.767	0.721	0.975	0.791	0.731	0.751
**AdaBoost**	Training	0.910	0.776	0.758	0.923	0.949	0.695	0.842
	Testing	0.818	0.800	0.781	0.933	0.893	0.750	0.823

### Evaluation and interpretation of machine learning optimal model

**Fig 5:**
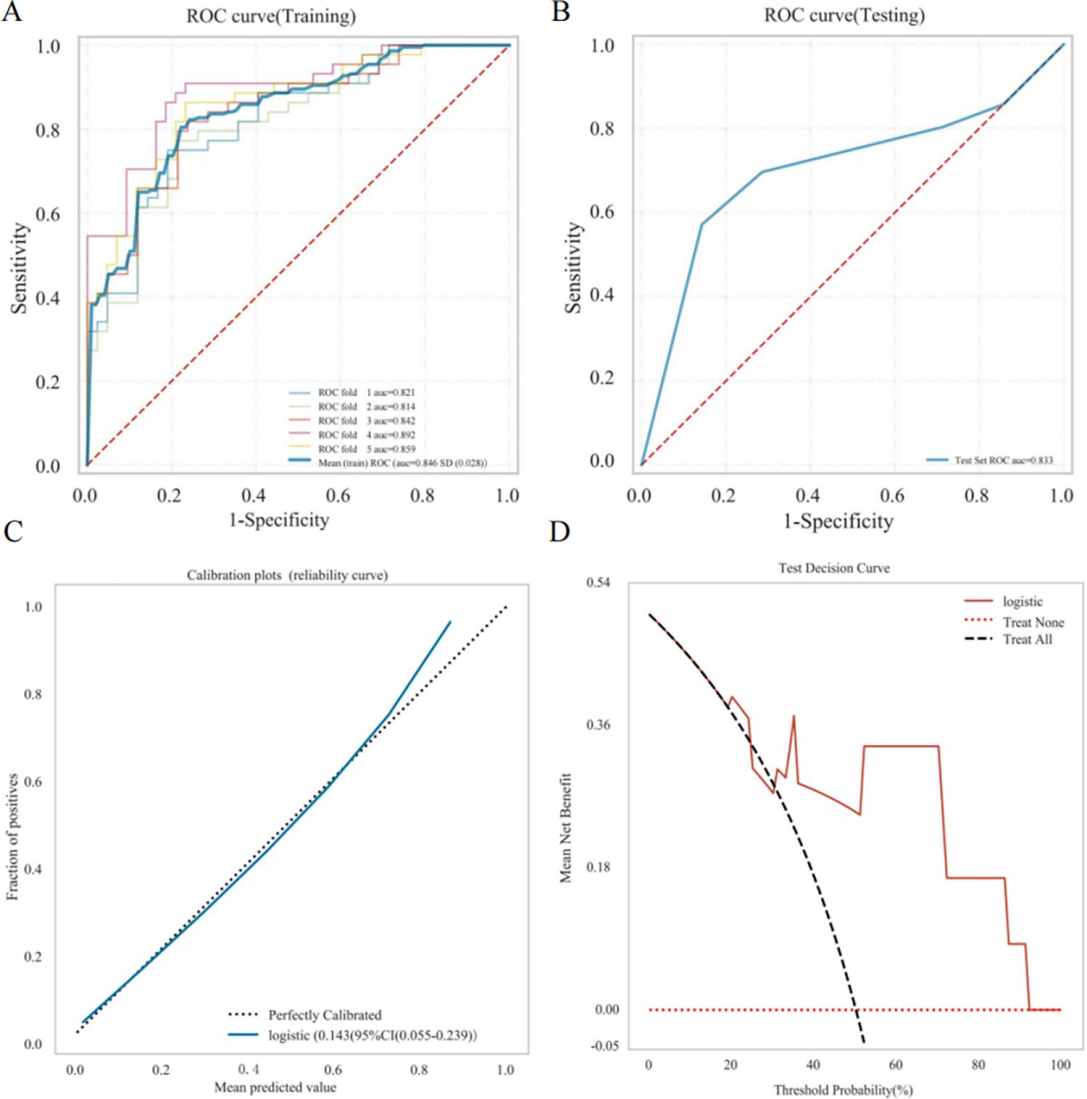
Diagnostic efficacy of the logistic regression model. (A) The training cohort’s ROC curve; (B) the testing cohort’s ROC curve; (C) Calibration curve; (D) Decision curve analysis.

As can be seen from Fig 5A and 5B, the logistic regression model exhibited strong diagnostic capabilities in predicting the recurrence of NPM. The calibration curve shown in Fig 5C revealed a significant correlation between the actual probabilities and the predicted probabilities, suggesting that the model is well-calibrated. Furthermore, Fig 5D showed the DCA curve, indicating that the model possesses considerable clinical utility.

**Fig 6:**
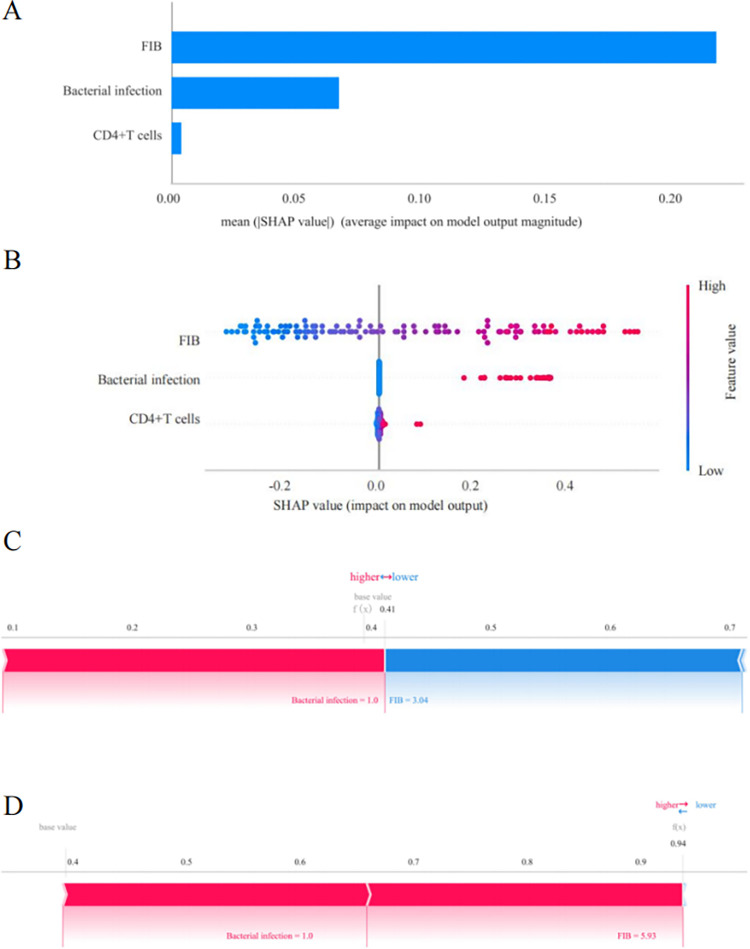
Visualization of the logistic regression model. **(**A) The SHAP analysis determined the ranking of the importance of various features. (B) The horizontal axis illustrates the SHAP value for each individual feature, measuring its precise impact on the final outcome. Each dot signifies a distinct sample. The color intensity, varying smoothly from red to blue, signifies the increasing or decreasing nature of the feature’s value, where red denotes a higher value while blue indicates a lower one. (C) The SHAP force plot for non-recurrent NPM patients. (D) The SHAP force plot for recurrent NPM patients.

According to the results in Fig 6A, the logistic regression model’s explanation, utilizing feature ranking from SHAP, suggested the importance of FIB, bacterial infection, and CD4^+^T cell count in the model. In Fig 6B, the connection between the observed values and SHAP values was shown for the three most relevant features. A higher Shapley value for a feature implies greater importance in predicting the model’s outcomes. Using the SHAP plot, the study visualizes the Shapley values of each feature, revealing their positive or negative impacts on the model. Fig 6C and 6D displayed the individual force plots for non-recurrent (Fig 6C) and recurrent (Fig 6D) NPM patients, respectively. Features with a positive impact are represented in red, meanwhile those with a negative impact are represented in blue. The longer the arrow, the greater the feature’s influence on the output. The scale values on the x-axis indicate the amount of decrease or increase in the impact.

### Conducting external validation of the logistic regression model

**Fig 7:**
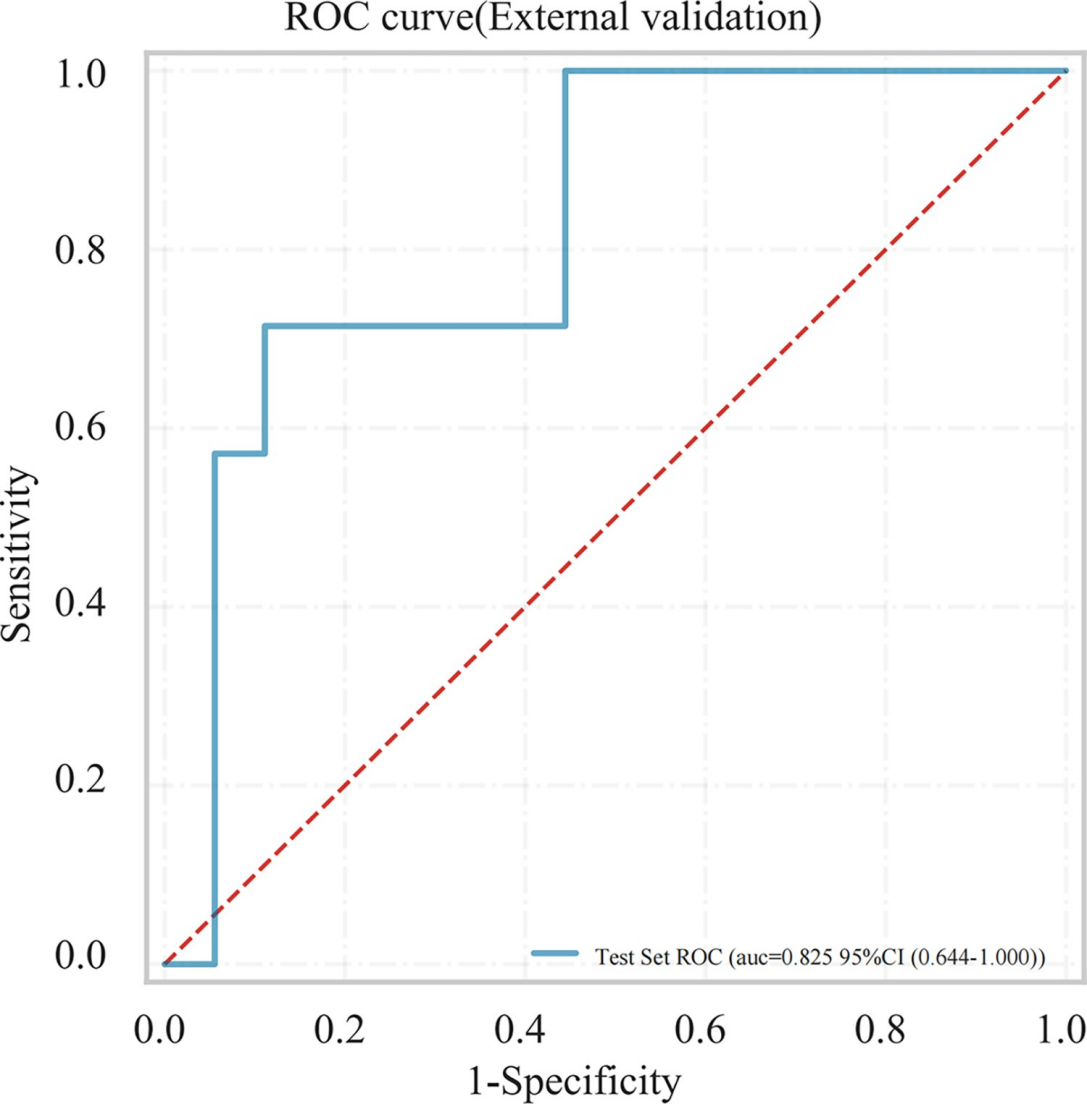
ROC for external validation of the logistic regression model.

An external validation set was constructed using data collected from 25 NPM patients from another center, consisting of 18 non-recurrent and 7 recurrent cases, a newly constructed model based on these 25 samples achieved an AUC of 0.825 (Fig 7). This demonstrated the excellent generalization ability of the model, indicating its reliability and practicality.

## Discussion

Non-puerperal mastitis (NPM) encompasses a category of benign breast conditions that arise in women during the non-puerperal stage, with the underlying causes remaining elusive. NPM is characterized by its diverse types, long duration, and high recurrence rate, especially in refractory non-puerperal mastitis, which is a key focus and challenge in clinical practice [21]. There are racial differences in the occurrence of NPM, with most cases reported in Middle Eastern countries (such as Turkey, Iran, Saudi Arabia, etc.) and Asian countries (such as China, South Korea, Japan, etc. [22,23]). Additionally, a higher proportion of Hispanic women are affected in Western countries [24]. Fattahi et al.’s study on NPM recurrence found that 79.17% of the participants were of Caucasian ethnicity, suggesting a possible association between this ethnicity and NPM recurrence [16]. Existing studies have shown that a history of oral contraceptive use, pregnancy, lactation, smoking, and bacterial infection are significantly associated with recurrence [25–27]. Patients may have a recurrence if they present with purulent nipple discharge, multicentric lesions, accompanying autoimmune diseases, a complaint of fistulas, the presence of nodular erythematosus, low vitamin B12 levels, menstrual irregularities, or require a combination of surgical and medical treatment to achieve complete remission [28–30]. So far, there are no clear treatment guidelines that can effectively reduce the recurrence rate of NPM. Studies have confirmed that early diagnosis can help prevent recurrences [26]. Rona et al. [31] consider residual lesions after combined steroid and surgical treatment as a risk factor for recurrence. Seyidli et al. [32] suggest that when using steroid hormones to treat NPM, high IgG4 concentrations in breast tissue are associated with recurrence, and therefore, other immunosuppressive drugs should be added after steroid therapy for maintenance. Presently, there are limited models available for predicting NPM recurrence, thus there is an urgent need to develop models that can predict whether NPM patients will experience recurrence, in order to facilitate early diagnosis and treatment by clinicians.

The patients in this study were concentrated in the age range of 28–34 years, and most had a history of childbearing. NPM patients with a history of childbearing had a higher recurrence rate than those without a history of childbearing, which is consistent with previous studies [3–6,33]. In addition, although there was no significant difference in BMI between the two patient groups, their BMI values exceeded the normal reference range. Previous studies have shown that a high BMI increases the incidence of NPM [34]. A total of 57 indicators, including bacterial culture, routine blood tests, reproductive hormone tests, immune-related tests, and coagulation tests, were analyzed in NPM patients. Thirteen indicators—including bacterial infection, WBC, NE, MO, PDW, NLR, PLR, CRP, IL6, CD4^+^T cell count, B cell count, FIB, and DD—showed significant differences. Elevated prolactin (PRL) levels have a significant impact on the incidence of NPM [35]. However, not all NPM patients exhibit abnormal PRL levels; some patients still have PRL levels within the normal range [36]. Huang Y et al. [37] discovered that a significant difference in PRL levels before and following treatment serves as an independent predictor of recurrence, with patients having high PRL levels after treatment being at a higher risk of recurrence. In this research, there was no difference in PRL levels between the non-recurrent and recurrent groups before treatment. This finding may suggest that there is no direct correlation between pre-treatment PRL levels and the risk of recurrence. Additionally, considering the relatively small sample size of our study, this could also be a factor contributing to potential bias in the results.

The recurrent group had significantly higher rates of bacterial infection, as well as elevated levels of WBC, NE, MO, PDW, NLR, PLR, CRP, IL-6, FIB, and DD compared to the non-recurrent group. Multiple studies have conclusively established that bacterial infection serves as an independent risk factor for recurrence among NPM patients [38–40]. Clinicians frequently use complete blood counts (CBCs) in their daily clinical practice, particularly in cases involving inflammatory disorders and during subsequent treatment evaluations. Lately, hematological indicators for example, PLR and NLR have been increasingly used as simple and inexpensive biomarkers to demonstrate systemic inflammation. PDW represents the platelet volume distribution width. In this study, the levels of WBC, NE, MO, PDW, NLR, and PLR were significantly elevated in the recurrent group, indicating a certain correlation with the development and progression of NPM. Consequently, these markers can serve as indicators of the disease to a certain degree. Multiple studies have confirmed that high levels of WBC, NE, MO, NLR, and PLR are predictive of a dismal prognosis in NPM [41–44]. However, some studies have also shown that the NLR and PLR results of recurrent and non-recurrent NPM patients are roughly the same. CRP is a non-specific inflammatory marker widely used in various diseases such as cardiovascular disease, autoimmune diseases, and infectious diseases. Current research indicates that CRP levels can serve as a biomarker for assessing the severity and prognosis of NPM [41–43,45]. IL-6 serves as a pivotal regulator of inflammatory responses, triggering the release of vasoactive substances, promoting the secretion of fibrinogen, and facilitating the production of CRP [46]. Therefore, there is a certain correlation between IL-6 and CRP, as well as FIB. Similar to CRP, IL-6 is associated with the progression of NPM [45]. FIB is a member of the acute-phase reactant protein family, and its elevated levels are a non-specific response seen in various diseases such as infections and aseptic inflammation. Velidedeoglu et al. demonstrated significantly higher levels of FIB in the recurrent group, which is consistent with our findings [43]. DD, which is a degradation product of fibrin, can also be elevated due to infections and tissue necrosis [47].

NPM is considered an autoimmune disease [48,49], and patients with non-puerperal mastitis exhibit varying degrees of immune dysfunction. According to Chen et al. [50], patients with non-puerperal mastitis have been reported to have increased levels of IgG, IgM, IgA, and C4, with a decrease in C3. Conversely, Xu et al. [51] indicated that levels of IgG, IgM, IgA, C3, and C4 were all elevated in NPM patients. Zheng et al. [52] further proposed that the imbalance in the immune microenvironment of NPM patients may be closely related to alterations in the ratios of Th1/Th2 and Th17/Treg, providing us with a new perspective to understand the immunopathogenesis of NPM. Therefore, we have placed special emphasis on the role of immune cells in the recurrence of NPM. Compared with the non-recurrence group, the number of CD4^+^ T cells and B cells in the recurrence group was significantly lower. NPM is considered an autoimmune disease [48,49], and patients with non-puerperal mastitis exhibit varying degrees of immune dysfunction. CD4^+^ T cells are essential immune cells within the human immune system, distinguished by the presence of CD4 molecules on their surface. Their detection results play a pivotal role in assessing the immune function of patients. Previous studies have confirmed that NPM patients have a reduction in CD4^+^ T cells in their peripheral blood [53]. Targeted therapy based on CD4^+^ T cell subsets represents a potential treatment direction for NPM, and immunomodulatory targeted therapy can provide new ideas for personalized diagnosis and treatment of NPM [52]. B cells are primarily associated with humoral immunity and can differentiate into plasma cells upon antigen stimulation, secreting antibodies (immunoglobulins), and participating in immune regulation, inflammatory responses, and hematopoiesis. Compared to patients in remission, there is no significant change in B cells in patients with active NPM [54]. However, no studies have yet examined the correlation between B cells and NPM recurrence.

Machine learning has the ability to infer complex patterns from large amounts of data, and its application in medicine has garnered significant attention [55,56]. One of the most promising applications of machine learning in the medical field lies in the widespread development of "personalized" medical diagnosis and interpretation [57]. The application of machine learning in laboratory analysis in medicine is equally exciting, given that laboratory tests serve as invaluable aids in clinical decision-making. As staff members of a clinical support department in a hospital, we have attempted to use the results of clinical laboratory tests with machine learning to establish a model that can assist clinicians in predicting whether NPM patients will experience recurrence in the future.

In this study, bacterial infection, FIB, and CD4^+^ T cell count were identified as the most important factors affecting NPM recurrence and were used for model development. The evaluation conducted using ROC, AUC, DCA, and calibration plot, demonstrated that the model possessed excellent discriminatory and calibration capabilities in predicting patients who would experience recurrent non-puerperal mastitis. The DCA plot indicated its good capability and high clinical utility. Moreover, the model exhibited effectiveness in both the test cohort, achieving an AUC of 0.833, and the external validation cohort, attaining an AUC of 0.825. To date, no one has utilized laboratory test results to establish a machine learning-based model for diagnosing NPM recurrence. Utilizing an AUC of 0.846, this study has established a model capable of discriminating NPM recurrence. In this paper, a model to distinguish NPM recurrence was established using logistic regression and laboratory test results, providing a new direction for clinicians to assess disease prognosis.

## Limitation

This study is a retrospective analysis with a small sample size and a case-control design, which limits the validity of its results. We only included 120 patients diagnosed with non-puerperal mastitis to establish the model and 25 patients to validate the model. In the future, we will include more patients and incorporate more hematological parameters to optimize and refine our model. Additionally, while we have established and validated the model using data from China, there is a lack of confirmation across different countries and ethnicities.

## Conclusion

In summary, this study constructed a recurrence prediction model for NPM based on machine learning. This not only helps clinicians better assess patients’ prognosis but also provides new insights into personalized diagnosis and treatment of NPM. The recurrence group had significantly higher rates of bacterial infection, as well as higher levels of WBC, NE, MO, PDW, NLR, PLR, CRP, IL-6, FIB, and DD compared to the non-recurrence group. In contrast, the recurrence group had significantly lower numbers of CD4^+^ T cells and B cells compared to the non-recurrence group. The top three indicators (FIB, bacterial infection, and CD4^+^ T cell count) were filtered and used to build the model. Four machine learning models (XGBoost, Logistic Regression, Random Forest, AdaBoost) were evaluated on the identical dataset to determine the recurrence of NPM. Among them, the logistic regression model shows the best predictive performance. In the training cohort of the logistic regression model, the AUC was 0.846, and in the test cohort, the AUC was 0.833. External validation of the model (AUC = 0.825) was carried out utilizing data sourced from another center, confirming its good discriminative and calibration abilities. This model can be applied in clinical practice to assist in the prediction of NPM recurrence.

## Supporting information

S1 Code (PKL)

S1 Data (XLSX)

## References

[1] Shi L, Wu J, Hu Y, Zhang X, Li Z, Xi PW, et al. Biomedical Indicators of Patients with Non-Puerperal Mastitis: A Retrospective Study. Nutrients. 2022;14(22):4816. doi: 10.3390/nu14224816 36432503 PMC9695051

[2] Fei Z, Xing-Chen S, Xing-Song T, Zhi-Gang Y, Surgery CSOB. Clinical practice guidelines for diagnosis and treatment of patients with non-puerperal mastitis: Chinese Society of Breast Surgery (CSBrS) practice guideline 2021. Chinese Medical Journal. 2021;134(15):1765–7. doi: 10.1097/CM9.0000000000001532 34039865 PMC8367070

[3] Mohammed AA. Mammary duct ectasia in adult females; risk factors for the disease, a case control study. Ann Med Surg (Lond). 2021;62:140–4. doi: 10.1016/j.amsu.2021.01.023 33520211 PMC7820305

[4] Li SB, Xiong Y, Han XR, Liu ZY, Lv XL, Ning P. Pregnancy Associated Granulomatous Mastitis: Clinical Characteristics, Management, and Outcome. Breastfeed Med. 2021;16(9):759–64. doi: 10.1089/bfm.2021.0023 33872053

[5] Cadena-Semanate RE, Estrella-Tapia LF, Contreras-Yametti FI, Contreras-Yametti JE, Salazar-Molina RD. Adalimumab in a patient with refractory idiopathic granulomatous mastitis: A case report. Breast J. 2021;27(1):99–102. doi: 10.1111/tbj.14050 33142352

[6] Velidedeoglu M, Papila Kundaktepe B, Mete B, Ugurlu S. Idiopathic granulomatous mastitis associated with erythema nodosum may indicate a worse prognosis. Int J Rheum Dis. 2021;24(11):1370–7. doi: 10.1111/1756-185X.14218 34514701

[7] Steuer AB, Stern MJ, Cobos G, Castilla C, Joseph KA, Pomeranz MK, Femia AN. Clinical Characteristics and Medical Management of Idiopathic Granulomatous Mastitis. JAMA Dermatol. 2020;156(4):460–4. doi: 10.1001/jamadermatol.2019.4516 31968055 PMC6990845

[8] Costa Morais Oliveira V, Cubas-Vega N, López Del-Tejo P, Baía-da-Silva DC, Araújo Tavares M, Picinin Safe I, et al. Non-lactational Infectious Mastitis in the Americas: A Systematic Review. Front Med (Lausanne). 2021;8:672513. doi: 10.3389/fmed.2021.672513 34422853 PMC8378399

[9] Parperis K, Costi E, Philippou S, Hadi M, Derk CT. >Efficacy of disease-modifying antirheumatic drugs in the treatment of granulomatous mastitis: a systematic review. Rheumatol Int. 2024;44(11):2371–9. doi: 10.1007/s00296-024-05719-w 39283511

[10] Yaghan R, Hamouri S, Ayoub NM, Yaghan L, Mazahreh T. A Proposal of a Clinically Based Classification for Idiopathic Granulomatous Mastitis. Asian Pac J Cancer Prev. 2019;20(3):929–34. doi: 10.31557/APJCP.2019.20.3.929 30912417 PMC6825786

[11] Luo W, Xu B, Wang L, Xiang L, Lai M, Zhang X, Liu X. Clinical characteristics and predictive factors of erythema nodosum in granulomatous lobular mastitis. Australas J Dermatol. 2021;62(3):342–6. doi: 10.1111/ajd.13640 34106462

[12] Parperis K, Achilleos S, Costi E, Vardas M. Granulomatous mastitis, erythema nodosum and arthritis syndrome: case-based review. Rheumatol Int. 2021;41(6):1175–81. doi: 10.1007/s00296-021-04820-8 33649961

[13] Sener Bahce Z, Aktas H. Patients with idiopathic granulomatous mastitis accompanied by erythema nodosum. Int J Clin Pract. 2021;75(4):e13928. doi: 10.1111/ijcp.13928 33305438

[14] Zhang M, Pu D, Feng D, Shi G, Li J. Rare and Complicated Granulomatous Lobular Mastitis (2000–2023): A Bibliometrics Study and Visualization Analysis. J Inflamm Res. 2024;17:3709–24. doi: 10.2147/JIR.S465844 38882188 PMC11179654

[15] Sarmadian R, Safi F, Sarmadian H, Shokrpour M, Almasi-Hashiani A. Treatment modalities for granulomatous mastitis, seeking the most appropriate treatment with the least recurrence rate: a systematic review and meta-analysis. Eur J Med Res. 2024;29(1):164. doi: 10.1186/s40001-024-01761-3 38475841 PMC10929103

[16] Fattahi AS, Amini G, Sajedi F, Mehrad-Majd H. Factors Affecting Recurrence of Idiopathic Granulomatous Mastitis: A Systematic Review. Breast J. 2023;2023:9947797. doi: 10.1155/2023/9947797 37794976 PMC10547579

[17] Zack JE, Garrison T, Trovillion E, Clinkscale D, Coopersmith CM, Fraser VJ, Kollef MH. Effect of an education program aimed at reducing the occurrence of ventilator-associated pneumonia. Crit Care Med. 2002;30(11):2407–12. doi: 10.1097/00003246-200211000-00001 12441746

[18] Yılmaz TU, Gürel B, Güler SA, Baran MA, Erşan B, Duman S, Utkan Z. Scoring Idiopathic Granulomatous Mastitis: An Effective System for Predicting Recurrence? Eur J Breast Health. 2018;14(2):112–6. doi: 10.5152/ejbh.2018.3709 29774320 PMC5939974

[19] Sun J, Shao S, Wan H, Wu X, Feng J, Gao Q, et al. Prediction models for postoperative recurrence of non-lactating mastitis based on machine learning. BMC Medical Informatics and Decision Making. 2024;24(1):106. doi: 10.1186/s12911-024-02499-y 38649879 PMC11036744

[20] Liu X, Chen Q. Expert Consensus on Traditional Chinese Medicine Diagnosis and Treatment of Granulomatous Lobular Mastitis (2021 Edition). Chinese Journal of Integrated Traditional and Western Medicine Surgery. 2022;28(5):597–602.

[21] Gurleyik G, Aktekin A, Aker F, Karagulle H, Saglamc A. Medical and surgical treatment of idiopathic granulomatous lobular mastitis: a benign inflammatory disease mimicking invasive carcinoma. J Breast Cancer. 2012;15(1):119–23. doi: 10.4048/jbc.2012.15.1.119 22493638 PMC3318163

[22] Zhou F, Liu L, Liu L, Yu L, Wang F, Xiang Y, et al. Comparison of Conservative versus Surgical Treatment Protocols in Treating Idiopathic Granulomatous Mastitis: A Meta-Analysis. Breast Care (Basel). 2020;15(4):415–20. doi: 10.1159/000503602 32982653 PMC7490657

[23] Martinez-Ramos D, Simon-Monterde L, Suelves-Piqueres C, Queralt-Martin R, Granel-Villach L, Laguna-Sastre JM, et al. Idiopathic granulomatous mastitis: A systematic review of 3060 patients. Breast J. 2019;25(6):1245–50. doi: 10.1111/tbj.13446 31273861

[24] Barra F, Centurioni MG, Gustavino C, Alessandri F, Ferrero S. Idiopathic Granulomatous Mastitis: The Importance of Summarizing the Heterogenous Evidence of the Current Literature. J Invest Surg. 2022;35(3):721–2. doi: 10.1080/08941939.2021.1901162 33779465

[25] Abbi B, Sanghavi N, Lanjewar S, Fineberg S, Xie X, Gupta A, et al. Clinical, histological features, and predictors of relapse in patients with idiopathic granulomatous mastitis. Medicine (Baltimore). 2023;102(44):e35679. doi: 10.1097/MD.0000000000035679 37933043 PMC10627657

[26] Tasci HI, Turk E, Erinanc OH, Erkan S, Gundogdu R, Karagulle E. Factors Affecting Recurrence of Idiopathic Granulomatous Mastitis. J Coll Physicians Surg Pak. 2022;32(2):161–5. doi: 10.29271/jcpsp.2022.02.161 35108784

[27] Yuan QQ, Xiao SY, Farouk O, Du YT, Sheybani F, Tan QT, et al. Management of granulomatous lobular mastitis: an international multidisciplinary consensus (2021 edition). Mil Med Res. 2022;9(1):20. doi: 10.1186/s40779-022-00380-5 35473758 PMC9040252

[28] Tian C, Han X, Liu Z, Lv X, Ning P. Management of Granulomatous Lobular Mastitis and Risk Factors Associated with Recurrence. World Journal of Surgery. 2022;46(11):2706–14. doi: 10.1007/s00268-022-06687-7 35963955

[29] Basim P, Argun D, Argun F. Risk Factors for Idiopathic Granulomatous Mastitis Recurrence after Patient-Tailored Treatment: Do We Need an Escalating Treatment Algorithm? Breast Care (Basel). 2022;17(2):172–9. doi: 10.1159/000517399 35707181 PMC9149487

[30] Hua C, Li F, Shi Y, Xu Y, Zhu M, Wang Y, et al. Long-Term Outcomes of Traditional Chinese Medicine in the Treatment of Granulomatous Lobular Mastitis: A Two-Year Follow-Up Study on Recurrence and New Occurrence Rates with Analysis of Risk Factors. J Inflamm Res. 2024;17:7389–99. doi: 10.2147/JIR.S485589 39429855 PMC11491064

[31] Rona G, Arifoglu M, Cetin K, Kundes MF. Relationship of post-treatment radiological findings with relapses in idiopathic granulomatous mastitis patients. North Clin Istanb. 2024;11(5):391–7. doi: 10.14744/nci.2023.32309 39431024 PMC11487317

[32] Seyidli C, Aydogdu YF, Buyukkasap C, Kozan R, Nasirov M, Dikmen K, et al. The role of tissue IgG4 levels in steroid therapy in patients with idiopathic granulomatous mastitis. Clin Exp Med. 2024;24(1):173. doi: 10.1007/s10238-024-01444-7 39069567 PMC11284177

[33] Uysal E, Soran A, Sezgin E. Factors related to recurrence of idiopathic granulomatous mastitis: what do we learn from a multicentre study? ANZ J Surg. 2018;88(6):635–9. doi: 10.1111/ans.14115 28749045

[34] Wei C, Wang X, Zeng J, Zhang G. Body mass index and risk of inflammatory breast disease: a Mendelian randomization study. Nutr Hosp. 2024;41(1):96–111. doi: 10.20960/nh.04746 37522462

[35] Sheybani F, Naderi HR, Gharib M, Sarvghad MR, Mirfeizi Z. Idiopathic granulomatous mastitis: Long-discussed but yet-to-be-known. Autoimmunity. 2016;49(4):236–9. doi: 10.3109/08916934.2016.1138221 26829298

[36] Cai R, Zhao J, Qiao Z, Li Y. Idiopathic granulomatous mastitis with normal prolactin level caused by risperidone. Asian Journal of Surgery. 2021;44(5):763–4. doi: 10.1016/j.asjsur.2021.02.026 33757729

[37] Huang Y, Wu H. A retrospective analysis of recurrence risk factors for granulomatous lobular mastitis in 130 patients: more attention should be paied to prolactin level. Annals of Palliative Medicine. 2021;10(3):2824–31. doi: 10.21037/apm-20-1972 33549007

[38] Co M, Cheng VCC, Wei J, Wong SCY, Chan SMS, Shek T, Kwong A. Idiopathic granulomatous mastitis: a 10-year study from a multicentre clinical database. Pathology. 2018;50(7):742–7. doi: 10.1016/j.pathol.2018.08.010 30389215

[39] Tan QT, Tay SP, Gudi MA, Nadkarni NV, Lim SH, Chuwa EWL. Granulomatous Mastitis and Factors Associated with Recurrence: An 11-Year Single-Centre Study of 113 Patients in Singapore. World J Surg. 2019;43(7):1737–45. doi: 10.1007/s00268-019-05014-x 31049604

[40] Tsai MJ, Huang WC, Wang JT, Wang MY, Lee YH, Lin SW, et al. Factors associated with treatment duration and recurrence rate of complicated mastitis. J Microbiol Immunol Infect. 2020;53(6):875–81. doi: 10.1016/j.jmii.2020.03.028 32327329

[41] Zhou Y, Wu J, Ma L, Wang B, Meng T, Chen H, Ye M. Differences and significance of peripheral blood interleukin-6 expression between patients with granulomatous lobular mastitis and those with benign breast tumors. Front Med (Lausanne). 2023;10:1273406. doi: 10.3389/fmed.2023.1273406 37817809 PMC10561106

[42] Li Q, Wan J, Feng Z, Shi J, Wei W. Predictive Significance of the Preoperative Neutrophil-lymphocyte Ratio for Recurrence in Idiopathic Granulomatous Mastitis Patients. Am Surg. 2023;89(12):5577–83. doi: 10.1177/00031348231161793 36880848

[43] Velidedeoglu M, Kundaktepe BP, Aksan H, Uzun H. Preoperative Fibrinogen and Hematological Indexes in the Differential Diagnosis of Idiopathic Granulomatous Mastitis and Breast Cancer. Medicina (Kaunas). 2021;57(7):698. doi: 10.3390/medicina57070698 34356979 PMC8303264

[44] Cetinkaya O A, Celik SU, Terzioglu SG, Eroglu A. The Predictive Value of the Neutrophil-to-Lymphocyte and Platelet-to-Lymphocyte Ratio in Patients with Recurrent Idiopathic Granulomatous Mastitis. Eur J Breast Health. 2020;16(1):61–5. doi: 10.5152/ejbh.2019.5187 31912016 PMC6939709

[45] Huang YM, Lo C, Cheng CF, Lu CH, Hsieh SC, Li KJ. Serum C-Reactive Protein and Interleukin-6 Levels as Biomarkers for Disease Severity and Clinical Outcomes in Patients with Idiopathic Granulomatous Mastitis. J Clin Med. 2021;10(10):2077. doi: 10.3390/jcm10102077 34066203 PMC8150275

[46] Kumari N, Dwarakanath BS, Das A, Bhatt AN. Role of interleukin-6 in cancer progression and therapeutic resistance. Tumour Biol. 2016;37(9):11553–72. doi: 10.1007/s13277-016-5098-7 27260630

[47] Li J, Zhou K, Duan H, Yue P, Zheng X, Liu L, et al. Value of D-dimer in predicting various clinical outcomes following community-acquired pneumonia: A network meta-analysis. PLoS One. 2022;17(2):e0263215. doi: 10.1371/journal.pone.0263215 35196337 PMC8865637

[48] Deng JQ, Yu L, Yang Y, Feng XJ, Sun J, Liu J, et al. Steroids administered after vacuum-assisted biopsy in the management of idiopathic granulomatous mastitis. J Clin Pathol. 2017;70(10):827–31. doi: 10.1136/jclinpath-2016-204287 28931582

[49] Liu Y, Sun Y, Zhou Y, Tang X, Wang K, Ren Y, He J. Sinomenine hydrochloride inhibits the progression of plasma cell mastitis by regulating IL-6/JAK2/STAT3 pathway. Int Immunopharmacol. 2020;81:106025. doi: 10.1016/j.intimp.2019.106025 31810886

[50] Chen F, Feng J, Gao Q, L L. Non breast-feeding mastitis patient peripheral immune function test and its clinical significance. Theory and Practice of Surgery. 2015;20(252–4).

[51] Xu R, Guo QQ, Yang LP, Lai ML, Tong L. Variations of peripheral blood autoantibody, immunoglobuliln, and complement levels in patients with non-lactational mastitis and their clinical significances. Nan Fang Yi Ke Da Xue Xue Bao. 2016;36(8):1157–9. 27578591

[52] Zheng B, Song J, Lu M, Chen C, Sun S. Current Research Describing the Role of CD4(+) T Lymphocyte Subsets in the Pathogenesis of Granulomatous Lobular Mastitis. J Invest Surg. 2022;35(10):1790–5. doi: 10.1080/08941939.2022.2090035 36075587

[53] Emsen A, Köksal H, Uçaryılmaz H, Kadoglou N, Artaç H. The alteration of lymphocyte subsets in idiopathic granulomatous mastitis. Turk J Med Sci. 2021;51(4):1905–11. doi: 10.3906/sag-2012-192 33862673 PMC8569769

[54] Uarylmaz H, Kksal H, Emsen A, Kadoglou N, Arta H. The Role of Regulatory T and B Cells in the Etiopathogenesis of Idiopathic Granulomatous Mastitis. Immunological Investigations. 2020;(12):1–11. 10.1080/08820139.2020.1832114 33034215

[55] Beam AL, Kohane IS. Big Data and Machine Learning in Health Care. Jama. 2018;319(13):1317–8. doi: 10.1001/jama.2017.18391 29532063

[56] Obermeyer Z, Emanuel EJ. Predicting the Future—Big Data, Machine Learning, and Clinical Medicine. N Engl J Med. 2016;375(13):1216–9. doi: 10.1056/NEJMp1606181 27682033 PMC5070532

[57] Rabbani N, Kim GYE, Suarez CJ, Chen JH. Applications of machine learning in routine laboratory medicine: Current state and future directions. Clin Biochem. 2022;103:1–7. doi: 10.1016/j.clinbiochem.2022.02.011 35227670 PMC9007900

